# Transcriptome Profiling of Archived Sectioned Formalin-Fixed Paraffin-Embedded (AS-FFPE) Tissue for Disease Classification

**DOI:** 10.1371/journal.pone.0086961

**Published:** 2014-01-30

**Authors:** Kensuke Kojima, Craig April, Claudia Canasto-Chibuque, Xintong Chen, Manjeet Deshmukh, Anu Venkatesh, Poh Seng Tan, Masahiro Kobayashi, Hiromitsu Kumada, Jian-Bing Fan, Yujin Hoshida

**Affiliations:** 1 Liver Cancer Program, Tisch Cancer Institute, Division of Liver Diseases, Department of Medicine, Icahn School of Medicine at Mount Sinai, New York, New York, United States of America; 2 Illumina, Inc., San Diego, California, United States of America; 3 Department of Hepatology, Toranomon Hospital, Tokyo, Japan; Duke-NUS, Singapore

## Abstract

**Background:**

Archived tissues from previously completed prospective trials represent invaluable resource for biomarker development. However, such specimens are often stored as sections on glass slides, in which RNA is severely degraded due to prolonged air exposure. We evaluated whether a proportion of archived sectioned formalin-fixed paraffin-embedded (AS-FFPE) tissues yield transcriptome profiles comparable to freshly cut (FC) FFPE tissues, which can be used for retrospective class prediction analysis.

**Methods:**

Genome-wide transcriptome profiles of 6 to 7-year-old AS-FFPE tissue sections (generated from 5 to 16-year-old blocks) of 83 hepatocellular carcinoma (HCC) and 47 liver cirrhosis samples were generated by using whole-genome DASL assay (Illumina) and digital transcript counting (nCounter) assay (NanoString), and gene signature-based prediction of HCC subclasses and prognosis was compared with previously generated FC-FFPE profiles from the same tissue blocks.

**Results:**

RNA quality and assay reproducibility of AS-FFPE RNA were comparable to intermediate to poor quality FC-FFPE samples (RNA Integrity Number: up to 2.50, R-square for technical replicates: up to 0.93). Analyzable transcriptome profiles were obtained in 64 (77%) HCC and 36 (77%) cirrhosis samples. Statistically more confident predictions based on random resampling-based method (nearest template prediction) were obtained in 37 (58%) HCC and 13 (36%) cirrhosis samples. Predictions made in FC-FFPE profiles were reproduced in 36 (97%) HCC and 11 (85%) cirrhosis AS-FFPE profiles. nCounter assay was tested in 24 cirrhosis samples, which yielded confident prediction in 15 samples (63%), of which 10 samples (67%) showed concordant predictions with FC-FFPE profiles.

**Conclusions:**

AS-FFPE tissues yielded poorer quality RNA and transcriptome profiles compared to FC-FFPE tissues. Statistically more confident class prediction was feasible in 37 of 83 HCC samples and 13 of 47 cirrhosis samples. These results suggest that AS-FFPE tissues can be regarded as a resource for retrospective transcriptome-based class prediction analysis when they are the only available materials.

## Introduction

Clinical deployment of transcriptome-based biomarker has been a challenging task due to multiple reasons [1]. The major obstacles include limited availability of clinical specimens necessary for extensive validation of the signatures, and the time and cost required to conduct prospective trials to establish their clinical utility. To overcome these issues and accelerate the process of clinical translation of molecular biomarkers, Simon et al. proposed “prospective-retrospective” studies that perform retrospective analyses of previously completed prospective study cohorts [2]. However, the only available specimens archived in association with such previously conducted prospective trials are often formalin-fixed, paraffin-embedded (FFPE) tissue sections on glass slides. It is well known that RNA extracted from archived FFPE tissue section is severely degraded due to oxidation, cross-linking, and other chemical modifications, which are enhanced by prolonged air exposure [3]–[5].

We have successfully utilized the cDNA-mediated annealing, selection, extension and ligation (DASL) assay [6]–[8] for transcriptome profiling of FFPE tissues when RNA is extracted within a few weeks after the sectioning to minimize air exposure and further RNA degradation [9]–[20]. However, it is unknown how archival of FFPE tissues in the form of sections affects the result of transcriptome-based molecular classification, and what proportion of archived FFPE tissue sections can be used for the molecular classification. To answer these questions, we systematically evaluated transcriptome-based disease classification by comparing gene-expression profiles generated from archived sectioned FFPE (AS-FFPE) tissues with previously generated profiles of freshly-cut FFPE (FC-FFPE) tissue sections from the same tissue blocks.

## Materials and Methods

### AS-FFPE Tissue Specimens

We analyzed AS-FFPE tissue sections (one to three 10 micron-thick slices for each sample) sectioned from 5 to 16-year-old FFPE tissue blocks and archived for additional 6 to 7 years on glass slides at room temperature with drying reagent in sealed bags. These are subsets of samples analyzed in our previous studies, in which RNA was isolated from FC-FFPE tissues: 83 out of 118 hepatocellular carcinoma (HCC) tissues used to determine molecular subclasses [17] and 47 out of 82 liver cirrhosis tissues used to identify a prognostic 186-gene signature [19], for which AS-FFPE tissue sections were available. The FFPE tissues were obtained and archived as part of routine clinical care, and the ethics committee of Toranomon hospital approved the project and waived the need of written informed consent from the subjects on condition that all samples be made anonymous as previously described [17], [19].

Total RNA was isolated as previously described [19]. Briefly, after deparaffinization with CitriSolve (Fisher), AS-FFPE tissue was lysed with the lysis buffer B [21] with proteinase K overnight. Total RNA was isolated by using Trizol reagent (Invitrogen) as previously described [19]. The quality of RNA was evaluated by Bioanalyzer (Agilent), and the traces and RNA Integrity Number (RIN) were compared to those of FC-FFPE RNA samples known to yield good, intermediate, or poor quality whole-transcriptome profiles (WGDASL HT Assay Guide 15018210D, Illumina) [6].

### Whole-transcriptome Profiling

Whole-transcriptome profiling was performed by using the HumanHT-12 whole-genome DASL beadarray ver. 4.0 (Illumina). Total RNA was converted to cDNA using biotinylated oligo-dT18 and random nonamer primers, followed by immobilization to a streptavidin-coated solid support. The biotinylated cDNAs were then simultaneously annealed to a set of assay-specific oligonucleotides based on content derived from the National Center for Biotechnology Information (NCBI) Reference Sequence Database (release 98). The extension and ligation of the annealed oligonucleotides generate PCR templates that are then amplified using fluorescently-labeled (P1) and biotinylated (P2) universal primers. The labeled PCR products are captured on streptavidin paramagnetic beads, to yield single-stranded fluorescent molecules which are then hybridized, via gene-specific complementarity, to the HumanHT-12 BeadChip, whereafter fluorescence intensity is measured for each bead. Hybridized chips were scanned by using iScan (Illumina) and raw measurements were extracted by Genome Studio software ver. 3.0 (Illumina).

As a quality measure of the expression profiles, we calculated proportion of gene probes with a “present” signal computed based on built-in negative control probes by the Genome Studio software (%P-call). We also computed inter-sample correlation to detect outlier poor quality profiles as previously described [19] based on the assumption that the global transcriptome landscape is similar in the same type of adult tissues (range of %P-call varies according to tissue type, stage of tissue/organ development, assay platform, etc.). Briefly, we created a “median” array profile as a hypothetical representative sample in a dataset by calculating median for each gene on the microarray. The outlier poor quality profiles were identified based on dissimilarity to the “median” array measured by Pearson correlation. In our previous studies, we observed that the inter-sample correlation sharply dropped at a certain %P-call value, indicating that a certain number of genes should be detected to capture transcriptome landscape to perform robust data analysis [19]. Raw scanned data were normalized by using the cubic spline algorithm [22] implemented in the Illumina Normalizer module of GenePattern genomic data analysis toolkit (www.broadinstitute.org/genepattern) [23].

### Focused Gene Signature Profiling

The prognostic 186-gene signature was implemented in the digital transcript counting (nCounter) assay (NanoString) [24], [25] as reported in our previous study [9]. Total RNA (200 to 400ng) were assayed on nCounter Digital Analyzer (NanoString) according to the manufacturer’s instructions. Data were normalized by scaling with geometric mean of built-in control gene probes after log transformation (base 2) for each sample.

### Bioinformatics and Statistical Data Analysis

Prediction of HCC subclasses (HCC subclass meta-analysis signature) [17] and liver cirrhosis survival (186-gene signature) [19] was performed by using the nearest template prediction algorithm [26] implemented in the Nearest Template Prediction module of GenePattern. A prediction with a confidence p-value <0.05 was regarded as statistically more confident prediction. FC-FFPE profiles are publicly available at NCBI Gene Expression Omnibus (GEO) database (www.ncbi.nlm.nih.gov/geo) with accession numbers GSE10186 and GSE10140 for the HCC and liver datasets, respectively. AS-FFPE profiles newly generated in the current study are available at GEO with accession number GSE46444.

## Results

### Quality of RNA Extracted from AS-FFPE Tissue

**Figure 1:**
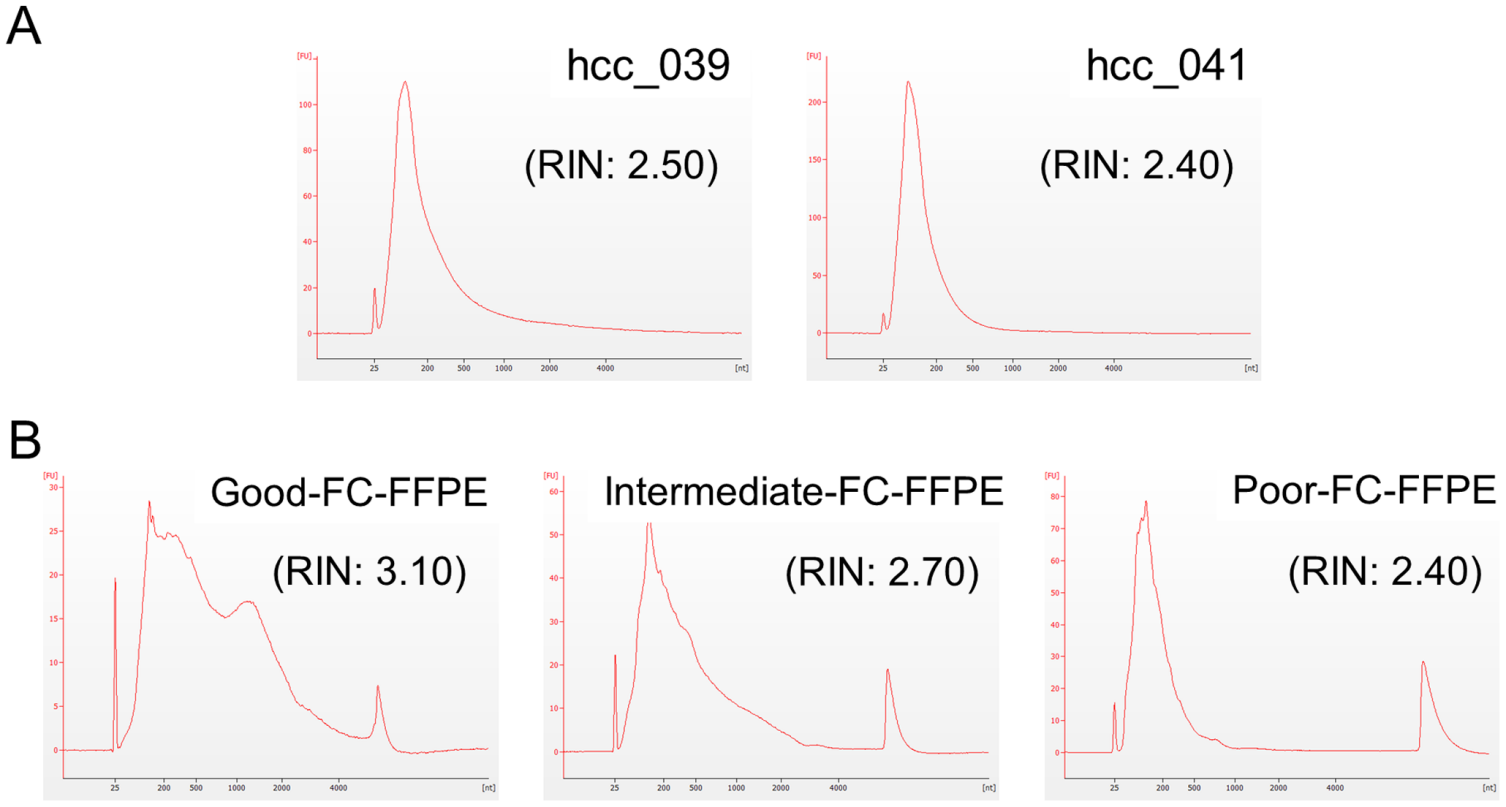
Bioanalyzer results for total RNA isolated from (A) AS-FFPE HCC tissues, and (B) good, intermediate, and poor quality FC-FFPE tissues. The AS- and FC-FFPE RNA samples were extracted from different samples. AS: archived section, FFPE: formalin-fixed paraffin-embedded, HCC: hepatocellular carcinoma, FC: freshly cut, RIN: RNA Integrity Number.

The quality of RNA extracted from AS-FFPE tissues was compared with FC-FFPE tissues. **Figure 1A** shows Bioanalyzer traces and RINs for two AS-FFPE RNA samples from HCC tissues. The peaks of the traces were observed around 100 bp, indicating that the majority of RNA fragments in the samples were approximately 100 bp long. RNA fragments longer than 1000 bp were rare. This result is comparable to the traces from FC-FFPE RNA samples known to yield intermediate to poor genome-wide transcriptome profiles (Illumina, WG-DASL systems manual) (**Figure 1B**). RINs were 2.40 and 2.50 for the analyzed AS-FFPE RNA samples, which are comparable to the RIN of poor quality FC-FFPE RNA.

### Quality of Whole-transcriptome Profiles of AS-FFPE RNA

**Figure 2:**
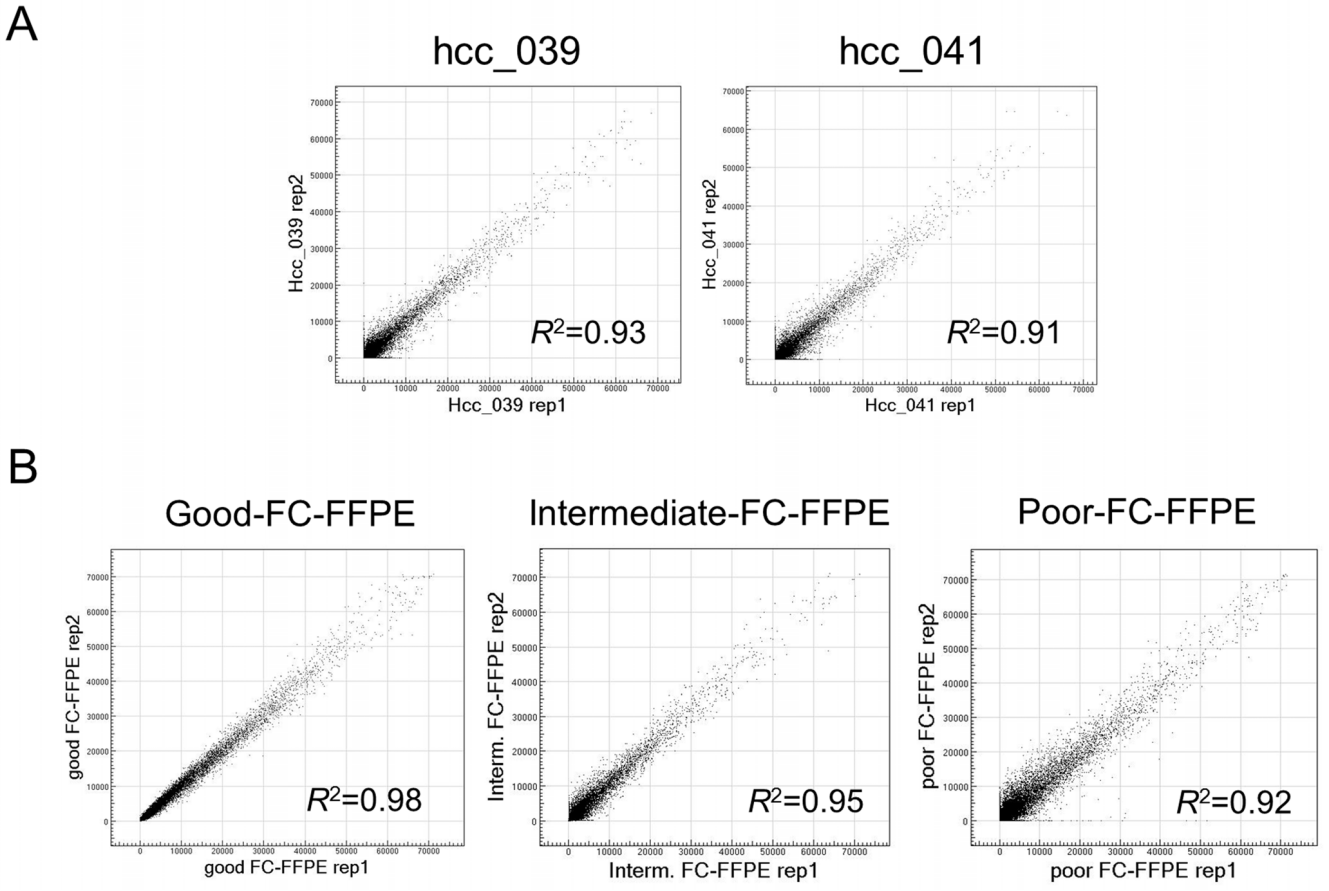
Reproducibility of genome-wide transcriptome profiles in (A) AS-FFPE HCC tissues, and (B) good, intermediate, and poor quality FC-FFPE tissues. Scatter plots of technical replicates are shown for each sample. The AS- and FC-FFPE RNA samples were extracted from different samples. AS: archived section, FFPE: formalin-fixed paraffin-embedded, HCC: hepatocellular carcinoma, FC: freshly cut.

**Figure 3:**
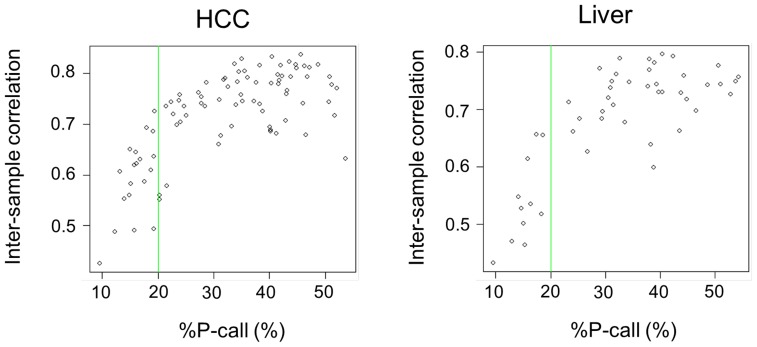
Quality of genome-wide transcriptome profiles of (A) AS-FFPE HCC and (B) liver cirrhosis tissues. Inter-sample correlation is plotted against %P-call. Green lines indicate quality cut-off of %P-call (20%). AS: archived section, FFPE: formalin-fixed paraffin-embedded, HCC: hepatocellular carcinoma, %P-call: % present call.

**Figure 4:**
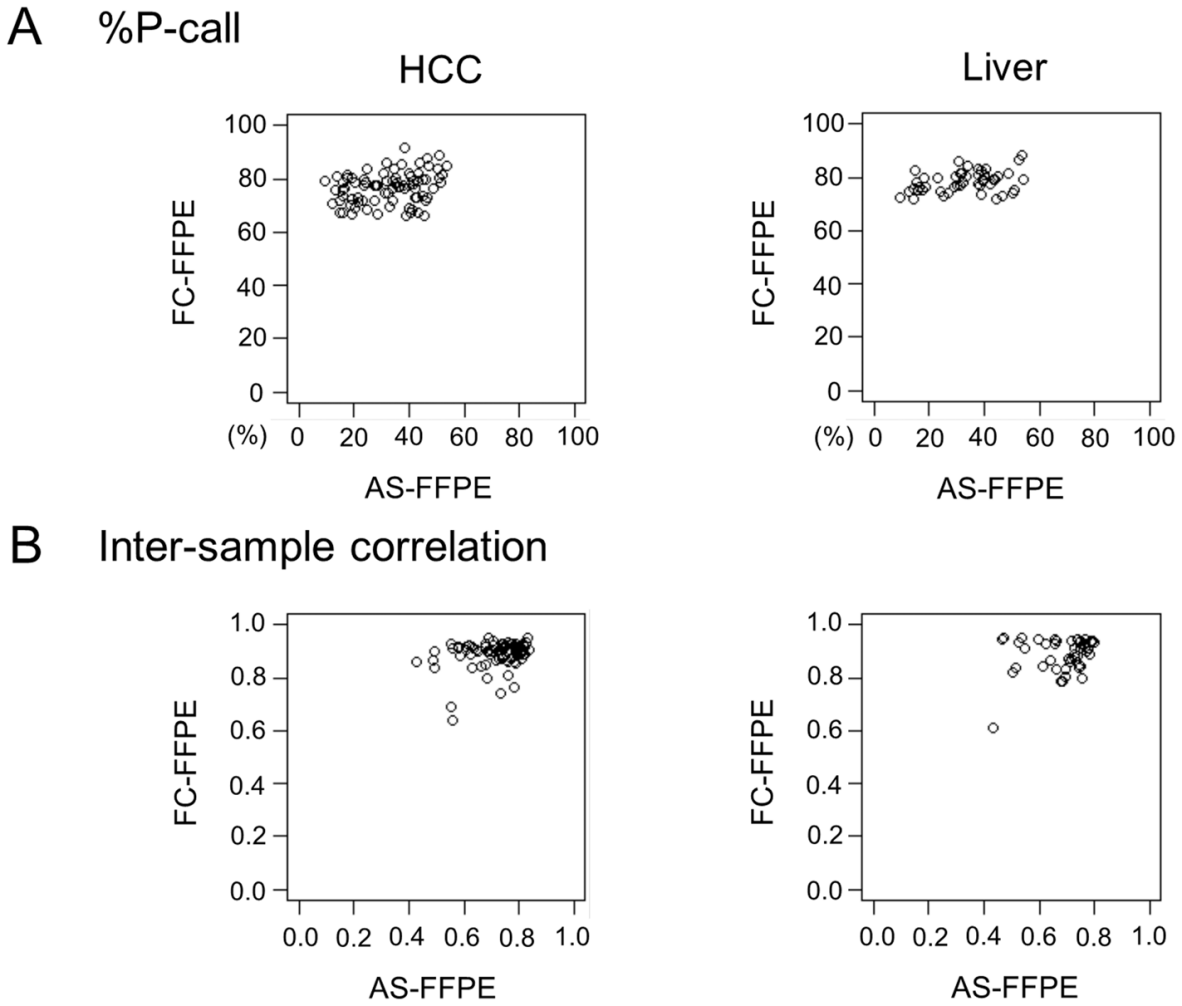
Comparison of (A) %P-call and (B) inter-sample correlation between AS-FFPE and FC-FFPE profiles. AS: archived section, FFPE: formalin-fixed paraffin-embedded, HCC: hepatocellular carcinoma, FC: freshly cut, %P-call: % present call.

Consistent with the Bioanalyzer result, whole-transcriptome profiling of technical replicates of AS-FFPE RNA showed reproducibility comparable to the intermediate to poor quality FC-FFPE RNA samples (**Figure 2**). Based on our previous study [6], the R^2^ values of 0.91–0.93 suggest that the amount of assayable RNA molecule was reduced to approximately 1/40 in AS-FFPE compared to FC-FFPE tissues. We next assessed how microarray signal detection is affected by archiving FFPE sections by comparing the proportion of probes with detected signal namely % present call (%P-call). It is expected that %P-call is similar and inter-sample correlation is high within adult tissues sharing the same tissue lineage [19]. More severe RNA degradation results in lower %P-call and inter-sample correlation. In the current datasets generated on the AS-FFPE samples, inter-sample correlation sharply dropped when less than 20% of the microarray probes detected gene expression signal (**Figure 3**). Based on this observation, we set the quality threshold of %P-call <20% to exclude poor quality profiles, and 64 out of 83 (77%) HCC and 37 out of 48 (77%) cirrhosis samples were further analyzed as having good quality transcriptome profiles. The values of the %P-calls are not directly comparable between the FC-FFPE and AS-FFPE profiles because they were generated using different assay platforms (transcriptionally informative 6k-gene array and whole-genome 29k-gene array, respectively). Nevertheless, the AS-FFPE profiles showed a wider range of %P-call values (10–60%) compared to the FC-FFPE profiles (70–90%) (**Figure 4A**). Similarly, the range of inter-sample correlations is also larger in AS-FFPE profiles (correlation coefficient of 0.4–0.9) compared to FC-FFPE profiles (0.6–1.0), suggesting more heterogeneous RNA quality in AS-FFPE tissues (**Figure 4B**). %P-call values for the AS-FFPE samples analyzed with Bioanalyzer were 44.8% (hcc_039) and 42.3% (hcc_041), and inter-sample correlations were 0.82 (hcc_039) and 0.79 (hcc_041), which were approximately in the middle of the ranges of %P-call and inter-sample correlation (**Figure 4**). This suggests that the RINs for these samples are representative of the AS-FFPE tissues. These results collectively demonstrate decay of transcriptome profiles in AS-FFPE tissues, which could affect retrospective gene signature-based class prediction.

### Transcriptome-based Class Prediction in AS-FFPE Profiles

**Figure 5:**
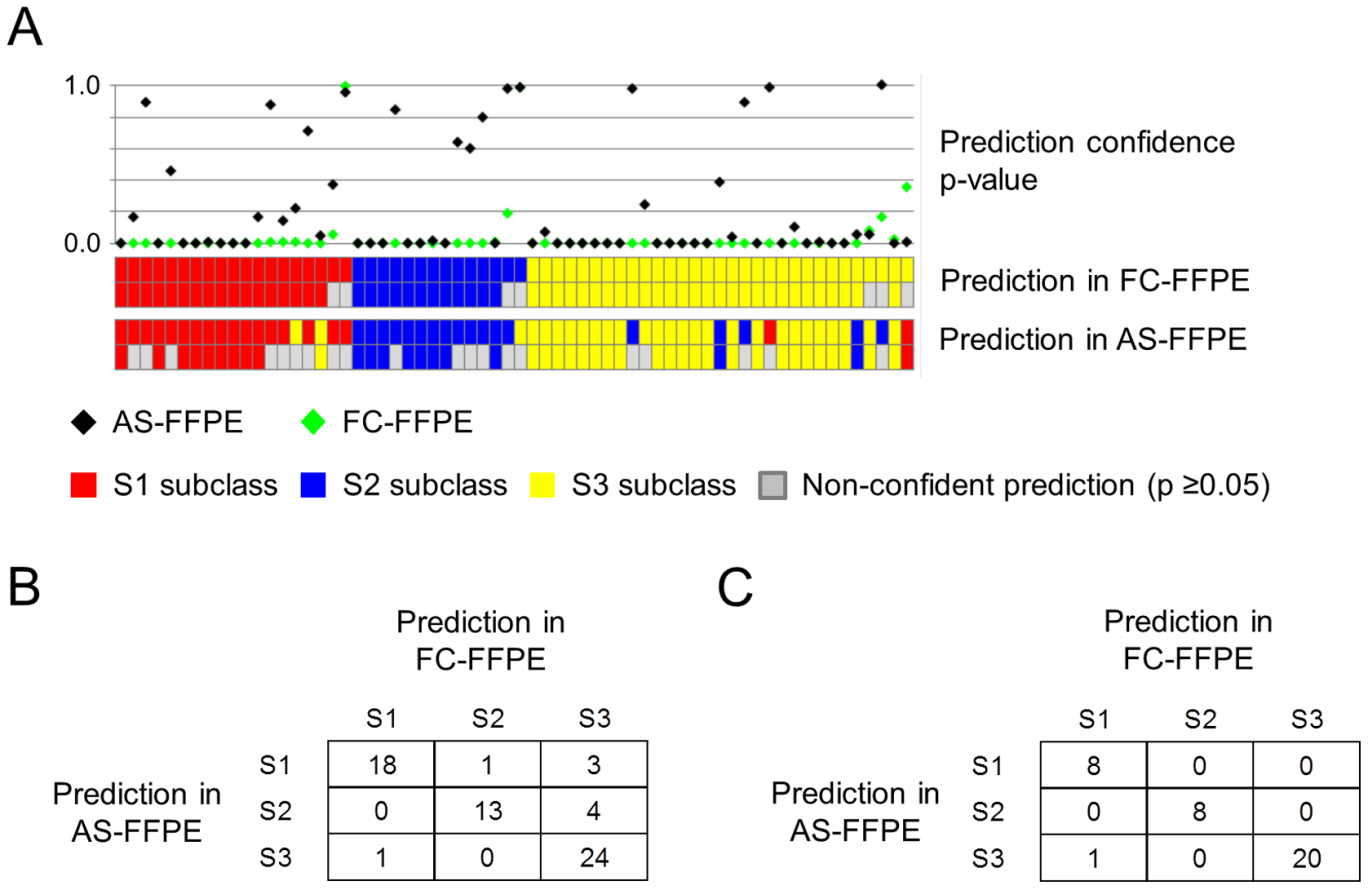
Comparison of transcriptome-based prediction of HCC subclass between FC-FFPE and AS-FFPE RNA extracted from the same tissue blocks (n = 64). (A) HCC subclass prediction result for each sample. Top panel indicates prediction confidence p-values for AS-FFPE (black diamond) and FC-FFPE (green diamond). Middle and bottom panels show prediction results made by using FC-FFPE and AS-FFPE profiles, respectively. (B) Contingency table of prediction results comparing FC-FFPE and AS-FFPE. (C) Contingency table of prediction results in 37 samples with statistically more confident prediction (prediction confidence p-value <0.05). AS: archived section, FFPE: formalin-fixed paraffin-embedded, FC: freshly cut, HCC: hepatocellular carcinoma.

We next evaluated gene signature-based class prediction in the AS-FFPE profiles in comparison to the FC-FFPE profiles. In the 64 HCC samples, a meta-analysis-based signature of three HCC subclasses (S1, S2, and S3), which has been well-validated in multiple independent cohorts and assay platforms [10], [13], [17], [27]–[29], was used to perform patient classification. Class prediction was performed by using previously developed model without making any modification (**Figure 5A**) [17]. Concordant prediction with FC-FFPE profiles was observed in 55 samples (86%) (**Figure 5B**). Statistically more confident predictions (p<0.05) for both FC-FFPE and AS-FFPE profiles were observed in 37 samples (58%), of which concordant prediction was observed in 36 samples (97%) (**Figure 5C**).

**Figure 6:**
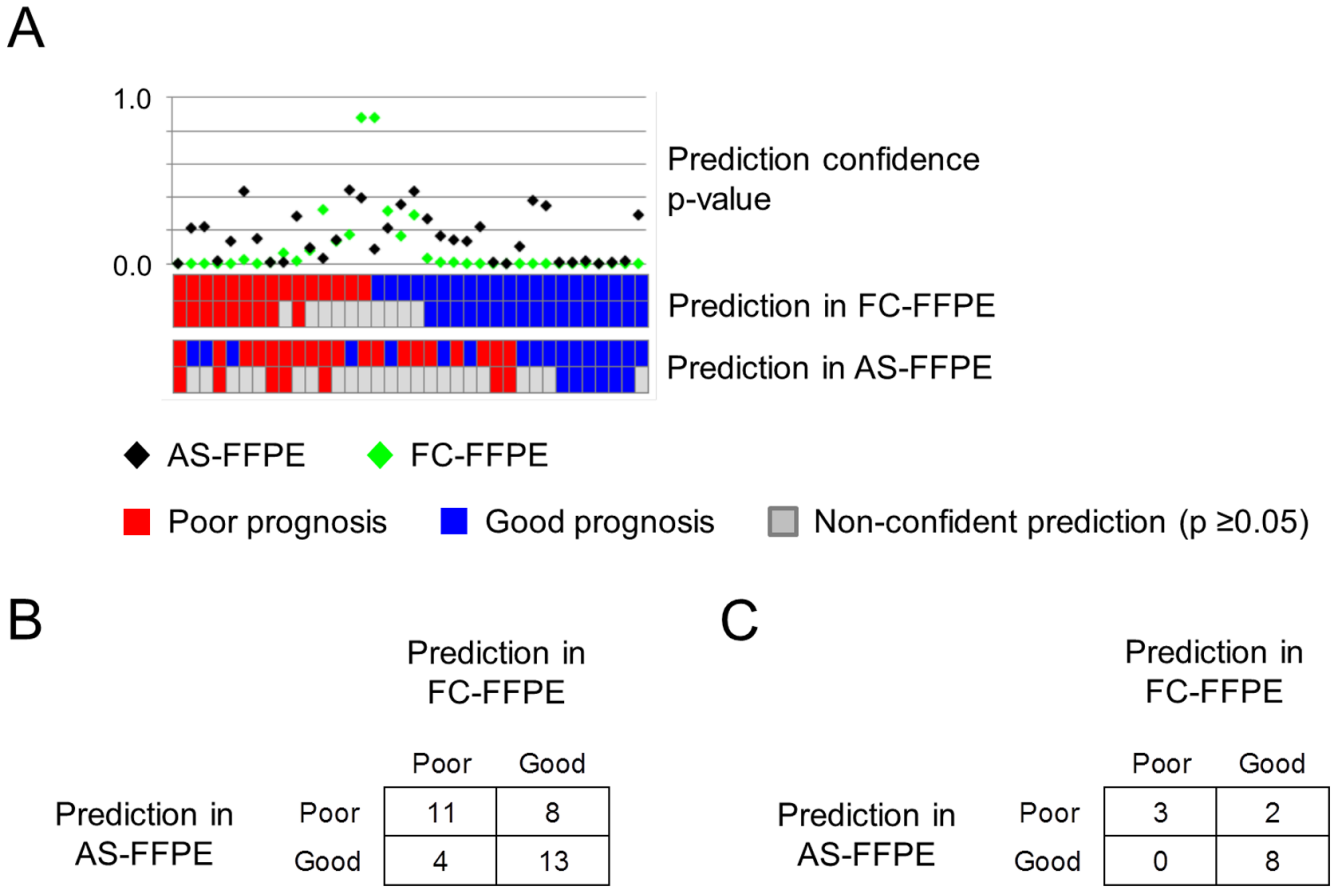
Comparison of transcriptome-based prediction of cirrhosis prognosis between FC-FFPE and AS-FFPE RNA extracted from the same tissue blocks (n = 36). (A) Cirrhosis prognosis prediction results for each sample. (B) Contingency table of prediction results comparing FC-FFPE and AS-FFPE. (C) Contingency table of prediction results in 13 samples with statistically more confident prediction (prediction confidence p-value <0.05). AS: archived section, FFPE: formalin-fixed paraffin-embedded, FC: freshly cut.

In the profiles of 36 liver cirrhosis samples, we performed class prediction using a prognostic 186-gene-expression signature, which has been extensively validated in multiple independent cohorts and assay platforms (**Figure 6A**) [9], [13], [19], [30]. Concordant prediction of poor or good prognosis was observed in 24 samples (67%) (**Figure 6B**). Statistically more confident predictions (p<0.05) for both FC-FFPE and AS-FFPE profiles were observed in 13 samples (36%), of which concordant prediction was observed in 11 samples (85%) (**Figure 6C**).

### AS-FFPE RNA Profiling with nCounter Assay

**Figure 7:**
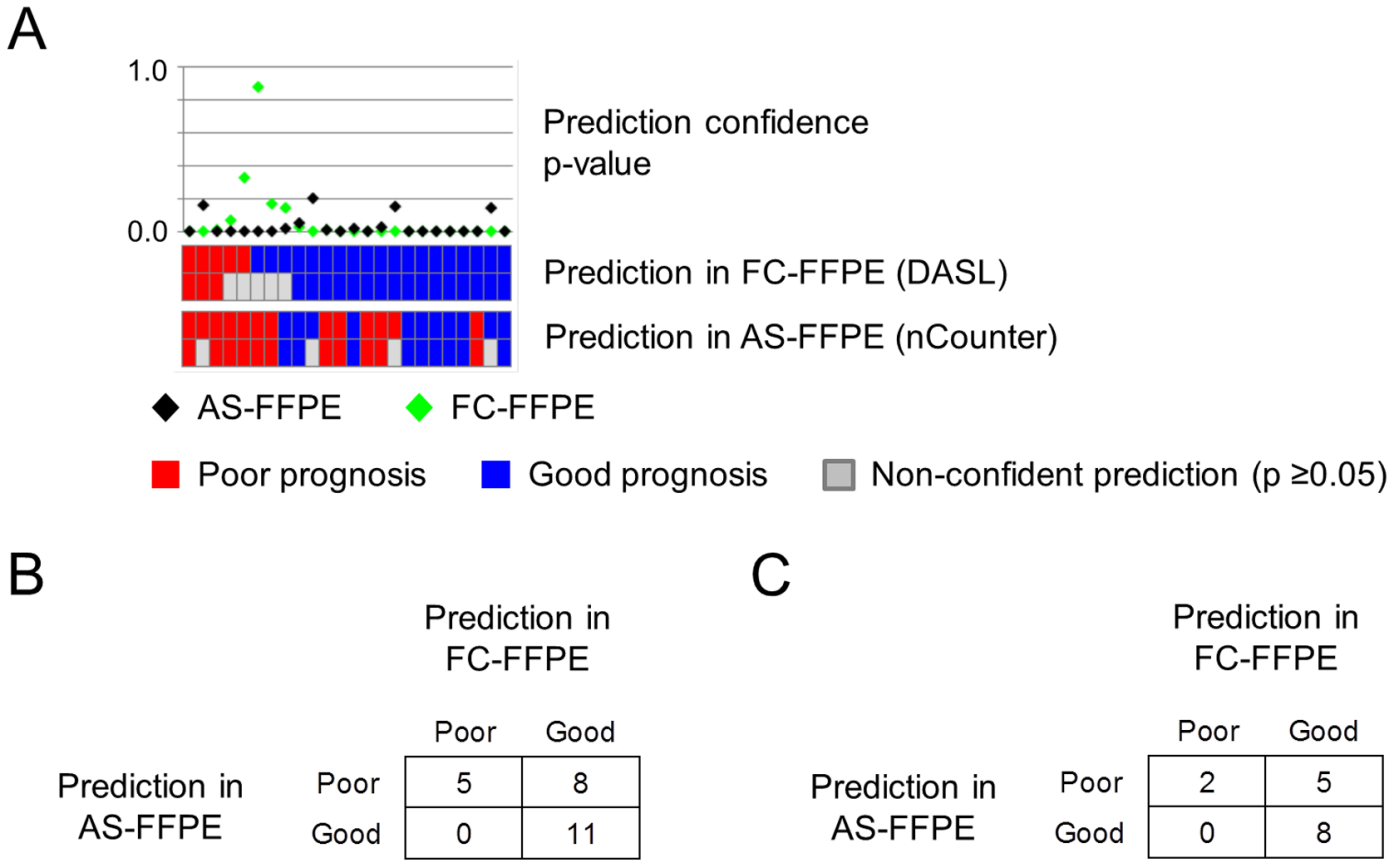
Comparison of transcriptome-based prediction of cirrhosis prognosis between FC-FFPE DASL and AS-FFPE nCounter profiles generated on the same tissue blocks (n = 24). (A) Cirrhosis prognosis prediction result for each sample. (B) Contingency table of prediction results comparing FC-FFPE and AS-FFPE. (C) Contingency table of prediction results in 13 samples with statistically more confident prediction (prediction confidence p-value <0.05). AS: archived section, FFPE: formalin-fixed paraffin-embedded, FC: freshly cut.

The 186-gene prognostic liver cirrhosis signature was assessed using the nCounter assay, which was specifically designed for clinical diagnostic lab and compatible with Clinical Laboratory Improvement Amendments (CLIA) certification [9]. RNA was extracted from 24 liver cirrhosis specimens, for which AS-FFPE tissues from the same block were available. Prognostic prediction was performed by using the 186-gene signature without making any modifications to the model and algorithm (**Figure 7A**). Concordant predictions with FC-FFPE were observed in 16 samples (67%) (**Figure 7B**). Twenty samples (83%) had statistically more confident predictions (prediction confidence p-value <0.05) for both FC-FFPE and AS_FFPE, of which 10 samples (67%) showed concordant prediction of poor or good prognosis (**Figure 7C**).

## Discussion

Recently developed genomic profiling technologies such as DASL have enabled analysis of archived FFPE tissue specimens with rich clinical annotations and follow-up necessary for validation of predictive/prognostic molecular signatures. However, these assays are optimized for FC-FFPE materials, and AS-FFPE is often the only available archived materials in many pathology labs and clinical trial study coordination centers. In addition, FC-FFPE tissue processing requires scheduling of sectioning and RNA isolation within a certain timeframe, which is limited by sample processing capability of each pathology lab. Therefore, analysis of AS-FFPE tissues will substantially increase the opportunities of retrospective assessment of predictive/prognostic molecular signatures.

Our results clearly demonstrated that the quality of AS-FFPE RNA is distinctly inferior to that of FC-FFPE RNA. Approximately three-fourths of AS-FFPE tissues yield analyzable transcriptome profiles for the purpose of retrospective class prediction analysis, and statistically more confident prediction was feasible in approximately one-third of the samples. The relatively good prediction confidence p-values and comparable prediction consistency in the nCounter assay suggest that the assay is another option to retrospectively analyze AS-FFPE tissues. The lack of target amplification may contribute to the higher prediction confidence. This may also indicate that high-throughput sequencing-based transcript counting could be an option once the assay is optimized for AS-FFPE specimens. A recent study showed minimal lab-to-lab and day-to-day experimental variations in the gene expression measurements in nCounter assay, further supporting its utility in the analysis of FFPE tissues [31].

The sites of degradation in FFPE RNA was observed roughly in random fashion in our previous study [19]. However, simultaneous analysis of multiple genes as a gene signature will prevent misclassification caused by missing signal from each single gene due to the sporadic RNA degradation. The successful application of this gene signature-based approach in our previous studies [9]–[20] strongly suggests that this is a viable option in analyzing real-world archived clinical specimens [32].

In summary, RNA isolated form AS-FFPE tissues was more degraded than that from FC-FFPE tissues, and yielded poorer quality transcriptome profiles. However, our results suggest that transcriptome signature-based disease classification is still feasible in a subset of AS-FFPE tissues, which will help increase the opportunities of retrospective validation and facilitate eventual clinical development of predictive/prognostic molecular signatures.
